# Editorial: Recent Advances in Gastrointestinal Cancers: From Microbiota Modulation to New Therapeutic Approaches

**DOI:** 10.3390/biomedicines13102540

**Published:** 2025-10-17

**Authors:** Serena Martinelli, Elena Niccolai

**Affiliations:** Department of Clinical and Experimental Medicine, University of Florence, 50139 Florence, Italy

Gastrointestinal (GI) cancers, including colorectal, pancreatic, and biliary tract malignancies, represent a major burden worldwide, characterized by high incidence, mortality, and clinical heterogeneity [1,2]. Despite advances in early detection and standard therapies, many patients present with refractory or aggressive disease, underscoring the urgent need for innovative therapeutic strategies, precise prognostic tools, and preclinical models that faithfully recapitulate human tumor biology.

The contributions included in this Special Issue reflect the multidimensional progress in the field, ranging from pharmacological innovations and biomarker discovery to patient-derived models and immune regulation, all framed within the evolving paradigm of precision oncology [3].

Advances in pharmacological management remain at the forefront. Regorafenib in combination with 5-fluorouracil (5-FU) has shown promising disease control and acceptable safety in heavily pretreated metastatic colorectal cancer (mCRC) [4]. Complementing this, real-world data suggest that the addition of bevacizumab to trifluridine–tipiracil (FTD-TPI) enhances progression-free survival and disease control rates, reinforcing the value of combinatorial strategies in mCRC clinical practice [5]. The efficacy of precision oncology relies also on robust molecular biomarkers for risk stratification and therapy guidance [6].

Similarly, multi-omics analyses in colon adenocarcinoma (COAD) identified hub genes, including Claudin1 (*CLDN1*), inhibin subunit beta A (*INHBA*), and chemokine (C-X-C motif) ligand 12 (*CXCL12*), as potential prognostic biomarkers, with single-cell RNA sequencing revealing cell-type-specific expression patterns [7]. In parallel, investigations into the Wnt/β-catenin pathway in colorectal adenomas underscore the importance of early molecular events and their translational relevance as biomarkers and therapeutic targets [8]. Together, these studies emphasize the integration of genomic, transcriptomic, and epigenetic data to advance precision medicine in GI oncology. Alongside these pharmacological and molecular advances, patient-derived organoids have become powerful platforms to evaluate therapeutic responses and interrogate disease mechanisms [9]. Organoids are three-dimensional, organ-like structures generated from self-organizing stem cells. They display organ-specific features and arise from stem cells undergoing intrinsic self-organization. Compared to traditional two-dimensional cell cultures, organoids offer significant advantages as they more closely replicate physiological cellular composition and function [10]. Organoid models derived from primary sclerosing cholangitis (PSC) and cholangiocarcinoma made possible the testing of JAK inhibitors and chemotherapy regimens while simultaneously exploring STAT3 expression in tumor and immune compartments [11]. Such models bridge the gap between molecular discoveries and clinical applications, providing a physiologically relevant context to predict patient specific responses [12]. Although not directly covered by the papers in this Special Issue, two emerging directions deserve attention for their potential to shape the future of GI oncology. The first is surgical innovation, particularly the development of intraoperative fluorescence-guided techniques. Indocyanine green (ICG) remains the most commonly used fluorophore in clinical practice for visualizing lymphatic drainage and facilitating sentinel lymph node mapping, yet its lack of tumor specificity represents a major limitation to its broader oncologic utility [13,14]. This limitation underscores the need for next-generation fluorescent contrast agents that selectively target tumor-specific surface biomarkers, thereby enabling more accurate intraoperative discrimination between malignant and healthy tissue [15]. Ongoing research is actively addressing this gap, aiming to develop targeted probes capable of enhancing both surgical precision and oncologic outcomes [9,16,17]. The second frontier, perhaps even more transformative, is the modulation of the gut microbiota. Altered microbial ecosystems are increasingly recognized as key players in colorectal carcinogenesis and tumor immunity. Recent studies have shown that microbiome profiling can discriminate between adenomatous polyps and colorectal cancer, highlighting microbial signatures as potential diagnostic and prognostic tools [18]. In parallel, advanced in vitro and in silico platforms are being developed to model the interactions between microbial communities, immune cells, and tumors, providing unprecedented opportunities to unravel mechanisms of immune modulation and treatment response [19]. The concept of immunonutrition, integrating diet, probiotics, and prebiotics into oncology care, is also gaining traction as a strategy to restore eubiosis, enhance immune surveillance, and synergize with pharmacological and immunotherapeutic interventions [20], and compelling evidence indicates that the gut microbiome shapes the efficacy and toxicity of chemotherapy and immunotherapy in GI cancers [21,22]. Approaches such as probiotics, prebiotics, dietary strategies, and even fecal microbiota transplantation are being actively investigated as adjunct therapies capable of overcoming resistance and potentiating antitumor immunity [23,24,25]. Taken together, the contributions in this Special Issue underscore the progress being made in GI oncology, particularly in pharmacological strategies, biomarker development, organoid technologies and immune regulation. At the same time, the exploration of surgical innovations and microbiota modulation exemplifies the dynamic expansion of the field toward a more holistic and personalized model of care. By integrating these avenues, precision oncology in GI cancers has the potential to significantly improve patient outcomes in the years to come.
